# Comparison of hepatic and serum lipid signatures in hepatocellular carcinoma patients leads to the discovery of diagnostic and prognostic biomarkers

**DOI:** 10.18632/oncotarget.23494

**Published:** 2017-12-13

**Authors:** Yonghai Lu, Juanjuan Chen, Chong Huang, Ning Li, Li Zou, Sin Eng Chia, Shengsen Chen, Kangkang Yu, Qingxia Ling, Qi Cheng, Mengqi Zhu, Weidong Zhang, Mingquan Chen, Choon Nam Ong

**Affiliations:** ^1^ Saw Swee Hock School of Public Health, National University of Singapore, Singapore; ^2^ Institute of Nutrition and Health, Qingdao University, Qingdao, Shandong, China; ^3^ School of Marine Science, Ningbo University, Ningbo, Zhejiang, China; ^4^ Department of Infectious Diseases and Hepatology of Huashan Hospital, Fudan University, Shanghai, China; ^5^ School of Pharmacy, Second Military Medical University, Shanghai, China; ^6^ NUS Environmental Research Institute, National University of Singapore, Singapore

**Keywords:** lipidomics, mass spectrometry, hepatocellular carcinoma, liver tissue, serum

## Abstract

We compared hepatic and serum lipid changes in hepatocellular carcinoma (HCC) patients to have a better understanding of the molecular pathogenesis of this disease and discovery novel lipid biomarkers. Hepatic and serum lipid profiling was conducted in paired liver and serum samples from 50 HCC patients and 24 healthy controls. A total of 20 hepatic and 40 serum lipid signatures were identified, yet there was hardly any significant correlation between them. The results indicated that triglycerides and phosphatidylcholines contributed significantly to altered hepatic lipids, whereas triglycerides and phosphatidylethanolamine-based plasmalogens (PEp) contributed most to altered serum lipids. In serum, PEp (36:4) and (40:6) showed a fair capability to discriminate HCC patients from healthy controls, and were significantly associated with HCC tumor grades (*p* < 0.05), and thus were identified as potential diagnostic and prognostic biomarkers of HCC. These findings were confirmed by a validation study conducted in an independent cohort consisting of 18 HCC, 20 cirrhosis patients, and 20 healthy controls. This study suggests that hepatic and serum lipid signatures of HCC have to be considered as mostly independent, and the results imply potential roles of PEp species, particularly PEp (36:4) and (40:6), as serum biomarkers for HCC diagnosis and progression.

## INTRODUCTION

Hepatocellular carcinoma (HCC) is one of the most frequently encountering malignancies, marked by a poor 5-year survival rate of less than 5%. In addressing the demands for molecular diagnosis and therapy of HCC, extensive efforts have been made in the last two decades to identify altered expressed genes by genomics, proteins by proteomics, and metabolites by metabolomics in HCC patients [1–6]. In addition, it is worth noting that numerous lipid changes in HCC patients were reported in metabolomics studies [6–14].

Lipids are a group of naturally occurring hydrophobic molecules that play important roles in biological functions by storing energy, signaling, and acting as structural components of cell membranes [15]. Recent advances in lipidomics provided a new angle for lipid investigation in cancer research [16–22]. Yet to date, however, only a few lipidomics studies have been carried out to characterize hepatic [23–25] and serum [26, 27] lipid profiles of HCC patients. Previous studies showed the increase of phosphatidylcholines (PCs) [23] and the reduction of phosphatidylethanolamines (PEs), phosphatidylserines (PSs) and phosphatidylinositols (PIs) [25] in HCC tumors. In serum, most lipids were found to be diminished in HCC patients compared to healthy controls and hepatitis patients, including PCs, sphingomyelins (SMs), triglycerides (TGs), and cholesterol esters (CEs) [26]. Building on these findings, our current knowledge of the aberrant lipid metabolism in HCC is still in its infancy, leaving much of the fundamental aspects of their biology unknown. Additionally, the relationship between hepatic and serum lipid changes in HCC patients remains unclear. Liver plays a key role in lipid metabolism, including synthesis of lipoproteins and intracellular catabolism of lipids [28]. The aberrant lipid metabolism in liver may provide first-hand information on the development of liver diseases. Blood serum is a readily accessible and widely used homeostatic biofluid in clinic tests, providing information in regard to an individual's physiological status [29]. Therefore, better understanding of the correlation between hepatic and serum lipid changes could improve the molecular characterization of HCC and help to identify disease-relevant biomarkers.

In this study, we characterized both hepatic and serum lipid profiles of 50 HCC patients using an untargeted lipidomics approach. When examining the lipid profiles, we aimed to compare the hepatic and serum lipid signatures in HCC patients, and also intended to identify the specific lipid biomarkers that could be used for diagnosis and progression of HCC.

## RESULTS

### Demographic and clinical characteristics of subjects

A total of 74 and 58 participants were respectively recruited from two study sites in this study, and their characteristics are shown in Table 1 and Supplementary Table 1. There were no significant differences on age and gender between groups in each batch. HCC patients had higher α-fetoprotein (AFP) levels than liver cirrhosis patients (*p* < 0.05) (Supplementary Table 1), and among HCC patients those who have cirrhosis also had higher AFP levels than those who do not have (Supplementary Table 2). Meanwhile, it was found that up to 40% (20 out of 50, Table 1) HCC patients had normal AF *P* values (i.e., < 20 mg/L) [30]. No significant differences were observed on alanine transaminase (ALT), aspartate transaminase (AST), and gamma-glutamyl transpeptidase (GGT) were found between HCC and liver cirrhosis patients (Supplementary Table 1), and between HCC patients with and without liver cirrhosis (Supplementary Table 2).

**Table 1:** Characteristics of 50 hepatocellular carcinoma (HCC) patients and 24 healthy individuals

Characteristics ^a^	HCC patients	Healthy individuals	*P* values ^b^
No. of subjects	50	24	
Gender (M/F)	38/12	19/5	0.766
Age (year)	53 (34∼72)	55 (24∼64)	0.706
AFP (mg/L)	45.3 (1.62∼24200)	-	
> 20/< 20	30/20		
ALT (U/L)	54 (17∼695)	-	
AST (U/L)	63.5 (20∼903)	-	
GGT (U/L)	86.5 (11∼647)	-	
HBsAg (positive/negative)	46/4	0/24	
HCVAb (positive/negative)	1/49	0/24	
Cirrhosis/no cirrhosis	29/21		
TNM stages			
T1N0M0 (early-stage)	12		
T2N0M0 (early-stage)	19		
T3N0M0 (late-stage)	10		
T4N0M0 (late-stage)	9		

^a^ Age, AFP, ALT, AST, and GGT in HCC patients were expressed as median (range). Limitation on the number of subjects, T1 and T2 were set as early-stage of HCC; T3 and T4 were set as late-stage of HCC.

^b^ Chi-square test for gender, and Student's t-test for age.

### Hepatic and serum lipid signatures in HCC patients

**Figure 1:**
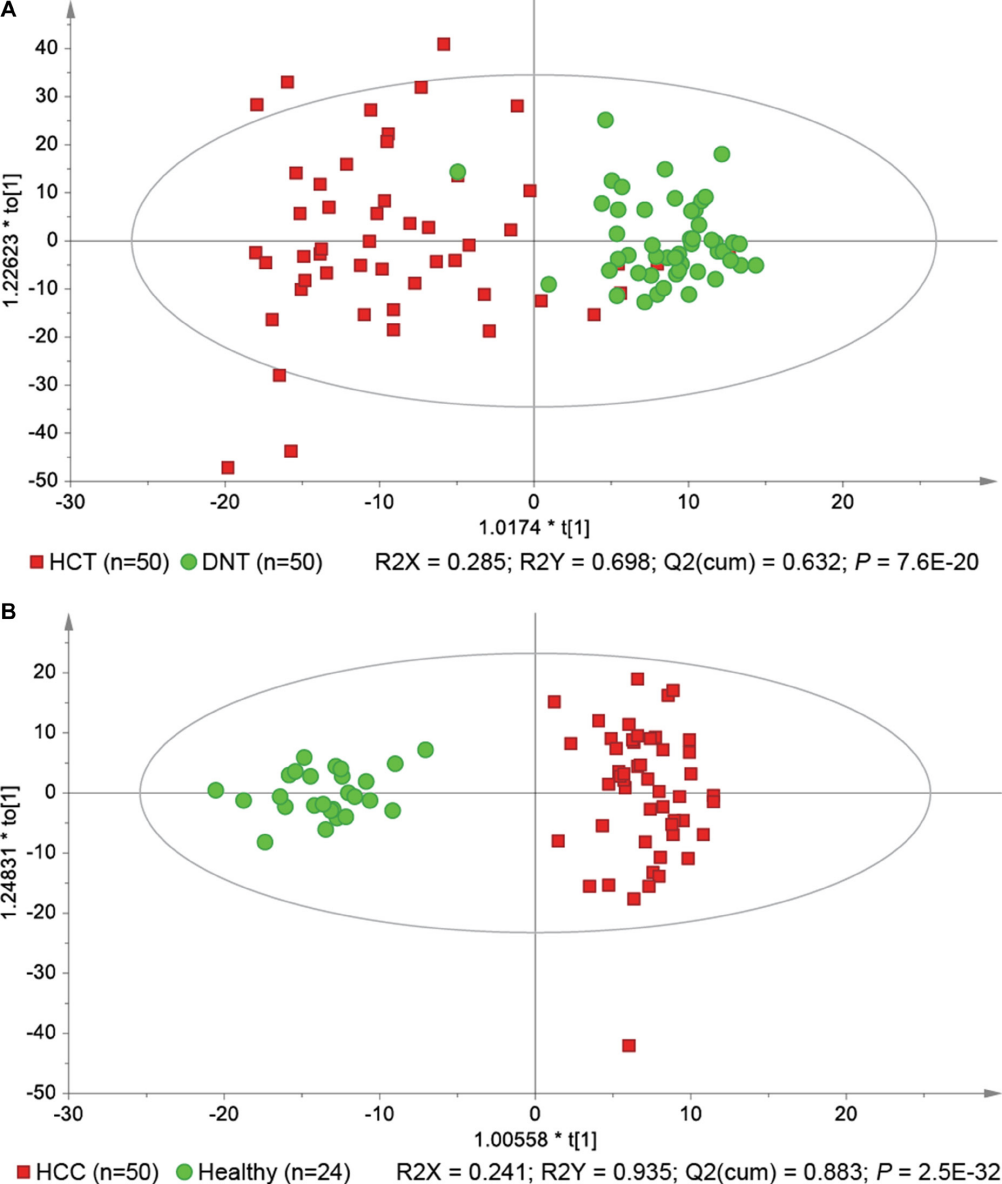
OPLS-DA score scatter plots show perturbations of hepatic (**A**) and serum (**B**) lipid profiles in hepatocellular carcinoma (HCC) patients.

We started our investigation using a principal component analysis (PCA) model to explore the variances of lipid profiles among the three types of liver tissues. As illustrated in the score scatter plot (Supplementary Figure 1), hepatocellular carcinoma tissue (HCT) samples were separated from adjacent noncancerous tissue (ANT) and distal noncancerous tissue (DNT) samples with a few overlaps, while there was no significant separation between ANT and DNT samples. Besides, we compared the lipid profiles between two respective groups among these three types of liver tissues, respectively. All the results demonstrated that lipid profiles were not significantly different between ANT and DNT samples. Thus, we focused on the differences between HCT and DNT samples to investigate hepatic lipid signatures of HCC. As previous report [6], orthogonal partial least-squares discriminant analysis (OPLS-DA) was applied to rank all lipids with respect to their performance for discriminating between HCT and DNT (Figure 1A). Finally, 20 lipid signatures were identified in HCC tumors, including six TGs, three PCs, three PEs, two phosphatidylglycerols (PGs), two PIs, two PSs, one SM, and one CE (Table 2). Their relative average quantities in HCT and DNT samples are shown in a heat map using MeV software (Supplementary Figure 2). Furthermore, OPLS-DA was applied to explore serum lipid signatures (Figure 1B). Finally, 40 lipid signatures were identified in serum, including 19 TGs, three lysophosphatidylcholines (LPCs), three PCs, eight PEs, one PI, three ceramides (Cers), one SM, and two CEs (Table 3). The relative average quantities of lipids in HCC patients and healthy controls are shown in Supplementary Figure 3. Stratification by cirrhosis status in HCC patients indicated that cirrhosis had no significant effects on the expression of most lipids in HCC patients (Supplementary Tables 3 and 4). On the basis of KEGG PATHWAY Database (http://www.genome.jp/kegg/) and literature, a map of HCC related lipid network was established based on these lipid signatures (Supplementary Figure 4).

**Table 2:** The 20 lipid signatures identified in the liver samples

Lipids	Candidates ^a^	R.T. (min)	m/z			Fragments	Formula	Trend ^b^
			[M+H]^+^	[M+NH4]^+^	[M-H]^-^			
**Glycerolipids**								
TG(51:0)	TG(16:0/17:0/18:0) [iso6]	15.96		866.8201		593, 579, 565, 267, 253, 239	C54H104O6	Up
TG(53:1)	TG(16:0/18:1/19:0) [iso6]	16.04		892.8365		619, 593, 577, 281, 265, 239	C56H106O6	Up
TG(55:2)	TG(15:0/18:1/22:1) [iso6]	16.12		918.8533		645, 619, 563, 321, 265, 225	C58H108O6	Up
TG(56:1)	TG(16:0/18:1/22:0) [iso6]	19.54		934.8825		661, 635, 577, 323, 265, 239	C59H112O6	Up
TG(58:1)	TG(16:0/18:1/24:0) [iso6]	22.98		962.9139		689, 663, 577, 351, 265, 239	C61H116O6	Up
TG(60:2)	TG(16:0/18:1/26:1) [iso6]	22.97		988.9299		715, 689, 577, 377, 265, 239	C63H118O6	Up
**Glycerophospholipids**								
PC(28:0)	PC(14:0/14:0)	6.69	678.5098			619, 468, 184	C36H72NO8P	Up
PC(31:0)	PC(15:0/16:0) [iso2]	7.89	720.5581			661, 494, 480, 184	C39H78NO8P	Up
PC(35:0)	PC(17:0/18:0) [iso2]	9.27	776.6212			717, 522, 508, 184	C43H86NO8P	Up
PE(34:2)	PE(16:0/18:2) [iso2]	7.61			714.5101	279, 255, 196	C39H74NO8P	Down
PE(38:6)	PE(16:0/22:6) [iso2]	7.38			762.5107	327, 255, 196	C43H74NO8P	Down
PE(40:6)	PE(18:0/22:6) [iso2]	8.06			790.5404	327, 283, 196	C45H78NO8P	Down
PG(36:3)	PG(18:1/18:2) [iso2]	6.51			771.5208	697, 281, 279, 153	C42H77O10P	Down
PG(36:4)	PG(18:2/18:2)	5.99			769.5057	695, 279, 153	C42H75O10P	Down
PI(34:2)	PI(16:0/18:2) [iso2]	6.63			833.5204	577, 553, 279, 255, 153	C43H79O13P	Down
PI(36:2)	PI(18:0/18:2) [iso2]	7.32			861.5523	581, 577, 283, 279, 153	C45H83O13P	Down
PS(38:6)	PS(16:0/22:6) [iso2]	6.83			806.4950	719, 327, 255, 153	C44H74NO10P	Down
PS(40:6)	PS(18:0/22:6) [iso2]	7.47			834.5307	747, 327, 283, 153	C46H78NO10P	Down
**Sphingolipids**								
SM(d34:2)	SM(d18:1/16:1)	6.75	701.5615			683, 184	C39H77N2O6P	Up
**Sterol lipids**								
CE(22:6)		11.75		714.6216		369	C49H76O2	Up

^a^ [iso6] means that the lipid might have six candidates/isomers, further analysis is needed for structure identification. Similar means for [iso2].

^b^ “Up” and “Down” represent the lipid is up- and down-regulated in HCC tumor tissues compared to matched normal tissues, respectively.

**Table 3:** The 40 lipid signatures identified in the serum samples

Lipids	Candidates^a^	R.T. (min)	m/z			Fragments	Formula	Trend ^b^	AUC ^c^
			[M+H]+	[M+NH4]+	[M-H]-				
**Glycerolipids**									
TG(44:1)	TG(12:0/14:0/18:1) [iso6]	11.07		766.6996		549, 521, 467, 265, 211, 183	C47H88O6	Down	0.76
TG(46:0)	TG(12:0/16:0/18:0) [iso6]	12.24		796.7466		579, 523, 495, 267, 239, 183	C49H94O6	Down	0.80
TG(46:1)	TG(12:0/16:0/18:1) [iso6]	11.59		794.7312		577, 521, 495, 265, 239, 183	C49H92O6	Down	0.80
TG(46:2)	TG(12:0/16:0/18:2) [iso6]	11.12		792.7155		575, 519, 495, 263, 239, 183	C49H90O6	Down	0.76
TG(46:3)	TG(12:0/16:0/18:3) [iso6]	10.79		790.6998		573, 517, 495, 261, 239, 183	C49H88O6	Down	0.65
TG(47:0)	TG(15:0/16:0/16:0) [iso3]	12.66		810.7620		551, 537, 239, 225	C50H96O6	Down	0.75
TG(48:0)	TG(16:0/16:0/16:0)	13.10		824.7780		551, 239	C51H98O6	Down	0.88
TG(48:1)	TG(14:0/16:0/18:1) [iso6]	12.27		822.7625		577, 549, 523, 265, 239, 211	C51H96O6	Down	0.81
TG(48:2)	TG(14:0/16:0/18:2) [iso6]	11.66		820.7471		575, 547, 523, 263, 239, 211	C51H94O6	Down	0.77
TG(48:3)	TG(14:0/16:1/18:2) [iso6]	11.36		818.7314		573, 547, 521, 263, 237, 211	C51H92O6	Down	0.68
TG(49:0)	TG(16:0/16:0/17:0) [iso3]	13.67		838.7937		565, 551, 253, 239	C52H100O6	Down	0.83
TG(49:1)	TG(16:0/16:1/17:0) [iso6]	12.70		836.7783		565, 563, 549, 253, 239, 237	C52H98O6	Down	0.84
TG(50:0)	TG(16:0/16:0/18:0) [iso3]	14.28		852.8092		579, 551, 267, 239	C53H102O6	Down	0.94
TG(51:1)	TG(16:0/17:0/18:1) [iso6]	13.72		864.8101		591, 577, 565, 265, 253, 239	C54H102O6	Down	0.85
TG(52:0)	TG(16:0/18:0/18:0) [iso3]	15.81		880.8416		607, 579, 267, 239	C55H106O6	Down	0.94
TG(52:1)	TG(16:0/18:0/18:1) [iso6]	14.33		878.8249		605, 579, 577, 267, 265, 239	C55H104O6	Down	0.93
TG(53:1)	TG(17:0/18:0/18:1) [iso6]	15.04		892.8420		605, 593, 591, 267, 265, 253	C56H106O6	Down	0.87
TG(53:2)	TG(17:0/18:1/18:1) [iso3]	13.76		890.8261		603, 591, 265, 253	C56H104O6	Down	0.86
TG(60:10)	TG(18:0/20:4/22:6) [iso6]	11.73		972.8104		671, 651, 627, 311, 287, 267	C63H102O6	Down	0.87
**Glycerophospholipids**									
LPC(18:3)		2.25	518.3275			500, 258, 184, 104	C26H48NO7P	Down	0.77
LPC(20:5)		1.86	542.3276			524, 258, 184, 104	C28H48NO7P	Down	0.72
LPC(22:6)		1.78	568.3458			550, 258, 184, 104	C30H50NO7P	Down	0.86
PC(33:0)	PC(15:0/18:0) [iso2]	6.74	748.5811			689, 522, 480, 184	C41H82NO8P	Down	0.80
PC(40:7)	PC(18:1/22:6) [iso2]	6.98	832.5880			773, 566, 520, 184	C48H82NO8P	Down	0.76
PC(40:9)	PC(18:3/22:6) [iso2]	7.13	828.5597			769, 566, 516, 184	C48H78NO8P	Down	0.75
PE(36:4)	PE(16:0/20:4) [iso2]	7.36			738.5105	303, 255, 196	C41H74NO8P	Down	0.79
PE(38:6)	PE(16:0/22:6) [iso2]	7.24			762.5046	327, 255, 196	C43H74NO8P	Down	0.81
PE(40:6)	PE(18:0/22:6) [iso2]	7.90			790.5393	327, 283, 196	C45H78NO8P	Down	0.80
PEp(36:4)	PE(P-16:0/20:4)	7.67			722.5156	436, 303, 259, 196	C41H74NO7P	Down	0.83
PEp(38:4)	PE(P-18:0/20:4)	8.33			750.5464	464, 303, 259, 196	C43H78NO7P	Down	0.75
PEp(38:6)	PE(P-16:0/22:6)	7.52			746.5135	436, 327, 283, 196	C43H74NO7P	Down	0.87
PEp(40:6)	PE(P-18:0/22:6)	8.19			774.5468	464, 327, 283, 196	C45H78NO7P	Down	0.82
PEp(40:7)	PE(P-18:1/22:6)	7.64			772.5317	462, 327, 283, 196	C45H76NO7P	Down	0.81
PI(36:4)	PI(18:2/18:2)	4.69			857.5140	577, 279, 153	C45H79O13P	Up	0.78
**Sphingolipids**									
Cer(d32:0)	Cer(d18:0/14:0)	8.04	512.5089			494, 282, 264, 252	C32H65NO3	Up	0.88
Cer(d38:0)	Cer(d18:0/20:0)	10.00	596.6013			578, 282, 264, 252	C38H77NO3	Up	0.70
Cer(d40:0)	Cer(d18:0/22:0)	10.50	624.6342			606, 282, 264, 252	C40H81NO3	Up	0.66
SM(d42:1)	SM(d18:1/14:0)	6.42	675.5510			657, 184	C37H75N2O6P	Down	0.89
**Sterol lipids**									
CE(18:1)		12.92		668.6399		369	C45H78O2	Down	0.71
CE(22:6)		11.44		714.6257		369	C49H76O2	Down	0.86

^a^ [iso6] means that the lipid might have six candidates/isomers, further analysis is needed for structure identification. Similar means for [iso3] and [iso2].

^b^ “Up” and “Down” represent the lipid is up- and down-regulated in serum of HCC patients compared to health individuals, respectively.

^c^ AUC value of ROC analysis between HCC patients and healthy subjects.

**Table 4:** Percentage of variances and main loadings (loading value > 0.5) explained by first two factors for hepatic and serum lipid changes in hepatocellular carcinoma (HCC) patients

% of Variance	Liver		Serum	
	Factor 1 (31.8%)	Factor 2 (14.6%)	Factor 1 (36.6%)	Factor 2 (14.9%)
Main Loadings	TG(51:0)	PC(31:0)	TG(46:3)	PEp(36:4)
	TG(53:1)	PE(40:6)	TG(48:0)	PEp(38:4)
	TG(55:2)	PS(40:6)	TG(48:1)	PEp(38:6)
	TG(56:1)		TG(48:2)	PEp(40:6)
	TG(58:1)		TG(48:3)	PEp(40:7)
	TG(60:2)		TG(49:1)	PC(40:7)
	PC(28:0)		TG(50:0)	
	PC(35:0)		TG(51:1)	
			TG(52:0)	
			TG(52:1)	
			TG(53:1)	
			TG(53:2)	

### Comparison of hepatic and serum lipid signatures

Among the hepatic and serum lipid signatures, only three lipids were common between them, they are PE (38:6), (40:6), and CE (22:6). Among them, PE (38:6) and (40:6) showed the same change trend in liver and serum, but CE (22:6) showed an opposite trend. Although TGs, PCs, PIs, SMs and CEs were changed in both liver and serum, these lipid species showed opposite trends. In addition, there was hardly any significant correlation between hepatic and serum lipid signatures in HCC patients (Supplementary Figure 5). These observations suggested that hepatic and serum lipid changes of HCC are rather difference and independent. With factor analysis (Table 4), it was found that TGs (factor 1) and PCs (factors 1 and 2) contributed most significantly towards the altered hepatic lipid metabolism in HCC patients, whereas TGs (factor 1) and phosphatidylethanolamine-based plasmalogens (PEp) (factor 2) contributed most significantly to the altered serum lipid metabolism.

### Diagnostic and prognostic capabilities of serum *lipid signatures*

**Figure 2:**
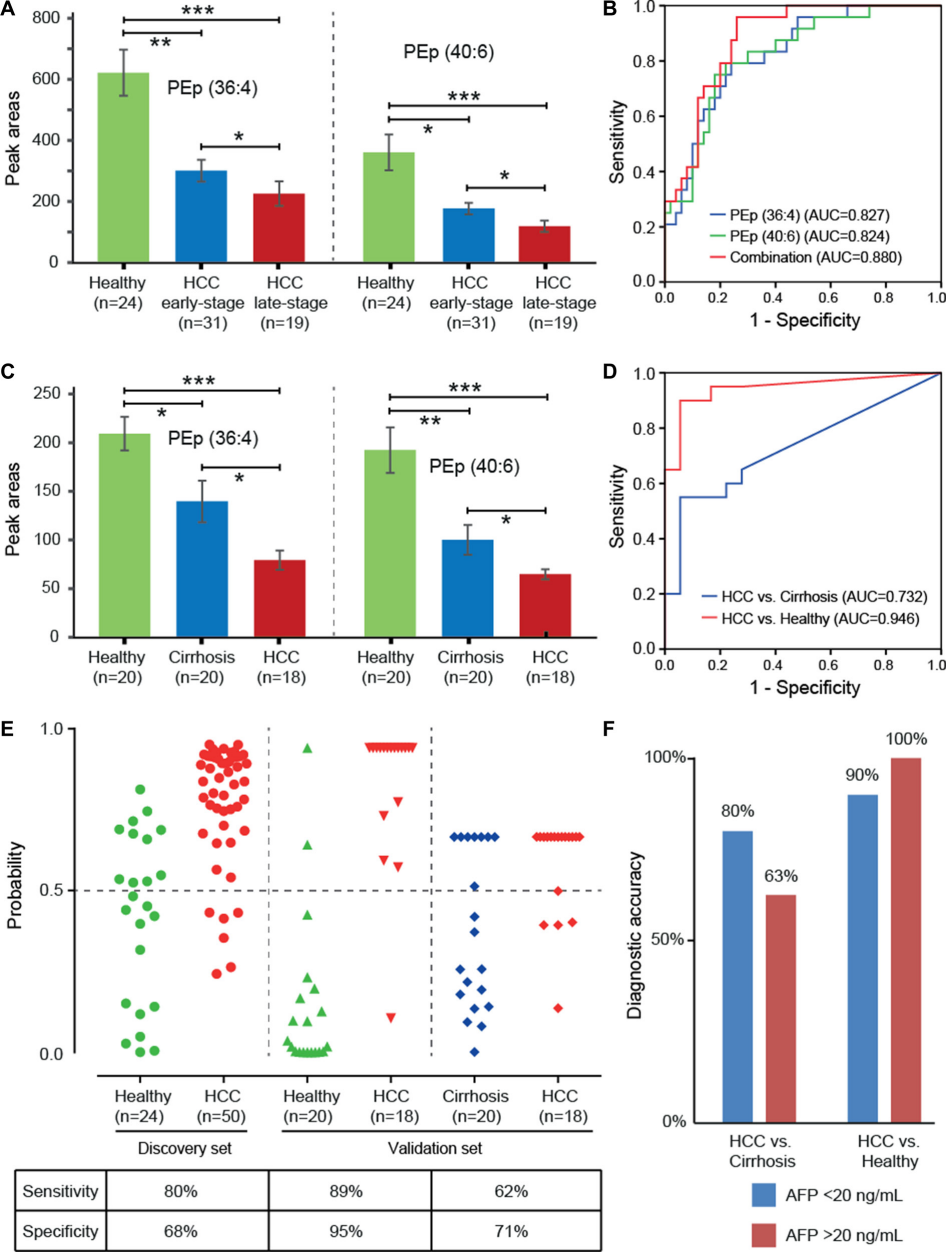
Diagnostic capabilities of PEp (36:4) and (40:6) for hepatocellular carcinoma (HCC) (**A**) Altered expressions of PEp (36:4) and (40:6) in serum of healthy subjects, early-stage HCC patients, and late-stage HCC patients; (**B**) ROC curve of serum PEp (36:4) and (40:6) in the discovery set; (**C**) Altered expressions of PEp (36:4) and (40:6) in serum of healthy subjects, liver cirrhosis patients, and HCC patients; (**D**) ROC curve of the combination of PEp (36:4) and (40:6) in the validation set; (**E**) Discrimination of healthy subjects, liver cirrhosis patients, and HCC patients by using the combined serum levels of PEp (36:4) and (40:6), at a cut-off of probability of 0.5; (**F**) Diagnostic accuracy of the combined marker PEp (36:4) and (40:6) for HCC patients with different concentrations of AFP in the validation set.

Receiver operating characteristic (ROC) analysis indicated that 24 out of 40 serum lipids showed a fair capability in diagnostic tests, with area under the curve (AUC) scores greater than 0.8 (Table 3). Of these, we found that PEp (36:4) and (40:6) showed a gradually decreased trend with the progression of HCC from early- to late-stages (Figure 2A), and their levels were associated with TNM tumor stage (*p* < 0.05) (Supplementary Table 5). Although our data showed that HCC patients with cirrhosis had lower levels of PEp (36:4) and (40:6) than those without cirrhosis (*p* < 0.05) (Supplementary Table 4), the decreased trend of these two lipids from early- to late-stages were not affected by the cirrhosis status, as the frequency of HCC patients suffering from cirrhosis was similar in the two stage groups, 60% and 58% respectively. Furthermore, a classification with AUC equal to 0.880 was achieved when using the two lipids together to classify HCC patients and healthy subjects (Figure 2B). Our results tend to suggest that PEp (36:4) and (40:6) appear to be potential serum biomarkers for diagnosis and progression of HCC.

### External validation

A semi-quantitative analysis of PEp (36:4) and (40:6) were carried out in the validation set to verify their capabilities for diagnosis and progression of HCC. Their levels were found to be significantly reduced in HCC and liver cirrhosis patients compared to healthy individuals, as well as their levels in HCC patients were much lower than those in liver cirrhosis patients (Figure 2C). Diagnostic tests showed that a combination of PEp (36:4) and (40:6) had good potential to discriminate HCC patients from both healthy individuals (AUC = 0.946) and liver cirrhosis patients (AUC = 0.732) (Figure 2D). Further, the c-statistic was increased from 0.732 in the basic model to 0.772 when putting AFP levels in the prediction model to differentiate HCC patients from liver cirrhosis patients (Supplementary Figure 6). The prediction probability values of the combined marker on HCC are shown in Figure 2E. At the cut-off value of 0.5 [31], 17 out of 18 HCC patients were correctly classified when compared to healthy individuals, giving a sensitivity of 89% and a specificity of 95%; 13 were correctly classified when compared to liver cirrhosis patients, giving a sensitivity of 62% and a specificity of 71%. It is worthy to note that the combination showed 80–90% diagnostic accuracy in these HCC patients with normal AF *P* values (< 20 ng/mL) (Figure 2F). Owing to the small samples size of HCC patients, we did not observer the significant association between PEp (36:4) and (40:6) levels with HCC tumor grade in the validation set (Supplementary Table 6), yet they still showed a gradually decreased trend (Supplementary Figure 7).

## DISCUSSION

In order to reveal the relationship between hepatic and serum lipid changes in HCC, we first characterized both hepatic and serum lipid profiles of 50 HCC patients and went further to identify the differential lipids. A total of 20 hepatic and 40 serum lipid signatures were identified in HCC patients, covering a wide range of lipid species including TGs, LPCs, PCs, PEs, PGs, PIs, PSs, Cers, SMs, and CEs. We noted that lipid metabolism in HCC tumors and sera are likely to be modulated in different manners, as most lipid species showed opposite tendency of changes in liver and serum, and only a few hepatic and serum lipids shared moderate correlations. Factor analysis confirmed that the altered hepatic lipid metabolism in HCC was mainly driven by TGs and PCs; the altered serum lipid metabolism was mainly driven by TG and PEp species.

Increased *de novo* lipogenesis is a common feature to most solid tumors, which occurs in tumourigenesis to supply energy and membrane lipids for accelerated cell proliferation [32]. TG is a precursor for synthesis of phospholipids, and also is a primary unit of energy storage [26], which is synthesized in liver beginning with acylation of glycerol-3-phosphate catalyzed by lysophosphatidic acid (LPA)-acyltransferase [33]. A recent study indicated that the isoleucine to methionine substitution at position 148 on patatin-like phospholipase domain-containing 3 (PNPLA3, adiponutrin) gene induced a gain of function in LPA-acyltransferase activity, leading to increased hepatic TG biosynthesis in HCC patients [34]. In addition, our previous study indicated that glycerol-3-phosphate significantly decreased in HCC tumors [6]. Taking them together, our current finding that TGs significantly increased in HCC tumors might be caused by the increased flux of glycerol-3-phosphate to TG in cancer cells. Besides *de novo* TG synthesis, HCC tumors also scavenge lipids from their environment. Previous study had indicated that uptake of fatty acids by cancer cells plays an important role in the development and progression of HCC [35]. Thus, enhanced TG uptake by HCC cancer cells from serum may also contribute to the increased TG levels in HCC tumors. It could also help explain why TG species reduced in serum of HCC patients as observed in our study and previous report [26], but further investigations are required to confirm these observations. Nevertheless, our study indicated an increase in TG synthesis/uptake in HCC tumors (Supplementary Figure 4).

Further, a series of glycerophospholipids were found to be altered significantly in HCC patients, including PCs, PEs, PGs, PIs, and PSs. Among them, PC and PE account for 40–60% of phospholipids of eukaryotic membranes and play important roles in cellular structure and biological functions [36]. A recent study showed that overexpression of LPC acyltransferase 1 enriched PC species in HCC tumors and promoted cell proliferation, which catalyzes the conversion of LPC to PC in the Lands cycle pathway of PC biosynthesis [24], which supports our finding that the elevated PC species in HCC tumors. In addition, we found that PC species were significantly decreased in serum of HCC patients, which was consistent with previous study as well [26]. In this study, five plasmalogens were found to be significantly reduced in serum of HCC patients compared to healthy controls, including PEp (36:4), (38:4), (38:6), (40:6) and (40:7). Plasmalogens are a type of ether phospholipids characterized by fatty alcohols with a vinyl ether linkage at sn-1 position and polyunsaturated fatty acids (PUFAs) with an ester linkage at sn-2 position. Although functions of plasmalogens have not yet been fully elucidated, it has been reported that they can reduce the damaging effects of reactive oxygen species in cancer cells [37]. To date, serum plasmalogen deficiencies have been linked to several cancers (e.g., colon cancer, prostate cancer, lung cancer, breast cancer, ovary cancer, and kidney cancer) [38].

Another important feature observed in HCC patients was the significant reduction of docosahexaenoic acid (DHA)-content glycerophospholipids, including LPC (22:6), PC (40:7), (40:9), PE (38:6), (40:6), PEp (38:6), (40:6), (40:7), and PS (38:6), (40:6) (Table 2 and 3). DHA is an omega-3 PUFA, which can be synthesized from alpha-linolenic acid, but mostly obtained directly from the diet. In 2009, Lim et al. found that omega-3 PUFAs such as DHA and eicosapentaenoic acid (EPA) could inhibit HCC cancer cell growth by blocking β-catenin and cyclooxygenase-2 [39]. The reduction of DHA-content glycerophospholipids observed in our study might be because of self-protection consumption, in which DHA was released from glycerophospholipids for self-care intervention. It is worth noting that our previous study also indicated a reduction in PUFAs (e.g., linoleic acid) in HCC tumors [6].

Ideally, the biomarkers used for disease diagnosis and progression should be easily accessible in clinical tests. Therefore, we only investigated the diagnostic and prognostic potential of serum lipid signatures for HCC. Although TGs, particularly TG (50:0), (52:0) and (52:1), showed the highest potential in the diagnosis of HCC with AUC > 0.9, their changes were not associated with the progression of HCC. Conversely, we found that the changes of PEp (36:4) and (40:6) were significantly correlated with TNM tumor grade (*p* < 0.05), and they also showed a moderate potential for HCC diagnosis (AUC >0.8). In this study, we aimed to find the lipid biomarkers with both diagnostic and prognostic capabilities, and thus we focused on the changes of PEp (36:4) and (40:6) in the validation study. The results confirmed that the two lipids could differentiate HCC patients form liver cirrhosis patients and healthy subjects with high sensitivity and specificity (Figure 2E). More importantly, they showed high diagnostic accuracy for HCC patients with low AF *P* values, providing a supplementary role to AFP for HCC diagnosis (Figure 2F).

The strengths of this study include the use of paired liver and serum samples and a well-established MS technique. This study is among the first to investigate the correlation between hepatic and serum lipid changes in HCC patients using an untargeted lipidomics method. Here, some limitations of our study still need to be acknowledged. First, the validation study was conducted within a limited number of participants. Further, in the validation phase, PEp (36:4) and (40:6) were only semi-quantitative because of no available standards. Therefore, the two candidate biomarkers from this study should be validated in a large replication cohort using standards.

In summary, a wide range of hepatic and serum lipid signatures was identified in HCC patients, including TGs, LPCs, PCs, PEs, PGs, PIs, PSs, Cers, SMs, and CEs. Compared with the limited lipid studies of HCC, our results indicated that hepatic and serum lipid changes in HCC patients might be modulated in different manners. The overall data indicated that PEp (36:4) and (40:6) showed significant diagnostic potential for HCC, and their levels were associated with TNM tumor grade. External validation verified that they are two candidate diagnostic biomarkers of HCC, while their potential roles as prognostic biomarkers of HCC need to be investigated further.

## MATERIALS AND METHODS

### Participants and sample collection

A batch of 50 HCC patients and 24 healthy volunteers were recruited at the Shanghai Huashan Hospital (Shanghai, China) between February 2011 and August 2012 (Table 1). Due to the limitation of subject numbers, 50 HCC patients were simply classified into early- and late-stages according to TNM staging system for progression investigation of HCC, in which T1 and T2 were set as early-stage of HCC (*n* = 31); T3 and T4 were set as late-stage of HCC (*n* = 19). Of these, 46 HCC patients were hepatitis B surface antigen (HBsAg) positive, and one was anti-hepatitis C virus (HCV) positive. Hepatic lipid profiling of 50 HCC patients was conducted by using a pairwise comparison of HCC tumor tissues and matched normal liver tissues of each patient. Three types of liver tissue were collected as surgical specimens from each HCC patient during surgery: HCT was from the central area of the solid tumor, ANT was collected at 1–2 cm surrounding the solid tumor, and DNT was collected at 5 cm away from the solid tumor. The tissue samples were snap-frozen with liquid nitrogen followed by storage at –80°C until analysis. Serum lipid profiling of 50 HCC patients was carried out by comparison with the 24 healthy subjects. The morning fasting antecubital venous blood samples were collected from the participants using a sterile 21-gage needle syringe, allowed to clot at 4°C overnight and centrifuged at 1,699 g for 10 min to obtain sera. The serum samples were stored in Eppendorf micro-tubes at –80°C until analysis. These 150 liver tissue and 74 serum samples were used as a discovery set for the identification of potential lipid signatures. The lipid signatures with diagnostic and prognostic potential were further evaluated in an independent batch of 58 serum samples from 18 HCC patients, 20 liver cirrhosis patients, and 20 healthy individuals, as described in the Supplemental Materials and Methods. The 58 subjects were recruited at the Jurong People's Hospital (Jiangsu, China) between April 2013 and June 2014 (Supplementary Table 1). Among them, 15 HCC and 14 liver cirrhosis patients were HBsAg positive, and 3 HCC and 6 liver cirrhosis patients were anti-HCV positive. All participants voluntarily joined this study, gave written informed consent, and completed a questionnaire that provided demographical information including age, gender, lifestyle factors, and medical family history. The study protocols were approved by the Institutional Review Boards at the Huashan Hospital, Jurong People's Hospital and National University of Singapore, and conducted in accordance with the Helsinki Declaration of 1975, as revised in 1996.

### Sample preparation

Tissue extraction was performed using a modified method to that described by Roberts et al. [40]. Frozen liver tissue (5 ± 0.05 mg) was mixed with 400 μL of chloroform/methanol (2:1, v/v) containing 2.5 μg/mL PC (15:0) and 5 μg/mL PC (17:0/17:0) as internal standards. The mixture was homogenized using a TissueLyser LT (Qiagen, UK) at 25 Hz for 10 min, then sonicated for 15 minutes. Following this, 200 μL of chloroform and 200 μL of water were added, then centrifuged for 10 minutes at 20,187 g. The organic phase was collected and filtered by Thermo Scientific™ national 750μL micro-centrifugal filters (PTFE membrane, 0.2 μm pore size, non-sterile). The filtrate was dried under nitrogen gas, then reconstituted in 200μL chloroform/methanol (2:1, v/v) for LC-MS analysis. Serum lipids were extracted using Bligh & Dyer's method [41] with minor modifications. Serum (100 μL) was diluted with 900 μL of chloroform/methanol/water (2:1:1, v/v/v) containing 2.5 μg/mL PC (15:0) and 5 μg/mL PC (17:0/17:0) as internal standards. The mixture was shaken vigorously for 5 min, and then centrifuged for 20 minutes at 20,187 g. The organic phase was collected and dried under nitrogen gas. The dry residues were reconstituted in 200 μL chloroform/methanol (2:1, v/v) for LC-MS analysis. Quality control (QC) samples were separately prepared for tissue and serum samples, and analyzed to evaluate the stability and reproducibility of LC-MS analytical system (Supplementary Figure 8).

### LC-MS analysis

Lipid profiling was performed on an Agilent 1290 ultrahigh pressure LC system (Waldbronn, Germany) coupled to a 6540 Q-ToF mass spectrometer equipped with an electrospray ionization (ESI) source. The samples were analyzed in both ESI-positive and -negative ion modes. The separation was performed on a phenomenex Kinetex C18 column (50 × 2.1 mm, 2.6 μm) at 40°C. The mobile phases were methanol/acetonitrile/isopropyl alcohol (9:4:2, v/v/v) with 20mM HCOONH_4_ (A) and water/isopropyl alcohol (13:2, v/v) with 20mM HCOONH_4_ (B). The gradient program was: 0–5 min, 60–85% A; 5–10 min, 85–100% A; 10–25 min, 100% A; 25–26 min, 100–60% A. The flow rate was set at 0.4 mL/min. A 10 μL of sample was loaded for each individual analysis. Mass data were acquired between m/z 100 and 1200 at a rate of two scans per second. The ion spray voltage was set at 4,000V, and the heated capillary temperature was maintained at 350°C. The drying gas and nebulizer nitrogen gas flow rates were 12.0 L/min and 50 psi, respectively. MS/MS analysis was carried out for the identification and characterization of lipid candidates with multiple collision energies, including 10, 20 and 40 V. Lipid identifications were conducted by searching the MS/MS data against entries in the Lipid MAPS (http://www.lipidmaps.org/) and METLIN (http://metlin.scripps.edu/) databases with mass errors of < 5 ppm. We named lipid species as combined the number of carbons and the number of double bonds, as well as the candidate isomers. A workflow of manual identification of lipids used in this study is shown in Supplementary Figure 9.

### Data pre-processing and statistical analysis

The hepatic and serum spectral data were exported as mzData files, and respectively pretreated by open-source software MZmine version 2.8 for peak detection, peak alignment and peak area normalization, in which the data of each sample was normalized to total area to correct for the MS response shift from the first injection to the last injection. The preprocessed lipid data were screened by using “80% rule” and missing values (i.e., zeros) were replaced by 1/2 minimum [42, 43] before statistical analysis. Analytical reliability of LC-MS method was assessed by a PCA model. The differential hepatic lipids in HCC patients were screened by comparing HCT and DNT samples. The lipids with variable importance in the projection (VIP) values of > 1.0 in the orthogonal partial least squares discriminant analysis (OPLS-DA) model and *p* values of < 0.05 in the paired t test were considered as signatures. Serum lipid signatures in HCC patients were defined by VI *P* values of > 1.0 in the OPLS-DA model and *p* values of < 0.05 in the Student's t test when compared to healthy subjects. Pearson correlation analysis were performed to reveal the significant correlation between hepatic and serum lipid changes. Factor analysis was applied to reduce dimensions of lipidomics data. ROC analysis was used to evaluate the diagnostic potential of lipids. SIMCA-P 14.0 software was used for multivariate statistical analyses (PCA and OPLS-DA). The paired *t* test, Student's t test, Pearson correlation analysis, factor analysis and ROC analysis were performed in IBM SPSS Statistics 24 software. *P* < 0.05 was considered significant. The false discovery rate method of Benjamini and Yekutieli was used to correct for multiple hypothesis testing & reduce false positives.

## SUPPLEMENTARY MATERIALS FIGURES AND TABLES



## Abbreviations

HCC: hepatocellular carcinoma
PCs: phosphatidylcholines
PEs: phosphatidylethanolamines
PSs: phosphatidylserines
PIs: phosphatidylinositols
SMs: sphingomyelins
TGs: triglycerides
CEs: cholesteryl esters
AFP: alpha-fetoprotein
ALT: alanine transaminase
AST: aspartate transaminase
GGT: gamma-glutamyl transpeptidase
PCA: principal component analysis
HCT: hepatocellular carcinoma tissue
ANT: adjacent noncancerous tissue
DNT: distal noncancerous tissue
OPLS-DA: orthogonal partial least-squares discriminant analysis
PGs: phosphatidylglycerols
LPCs: lysophosphatidylcholines
Cers: ceramides
PEp: phosphatidylethanolamine-based plasmalogens
ROC: receiver operating characteristic
AUC: area under the curve
LPA: lysophosphatidic acid
PNPLA3: patatin-like phospholipase domain-containing 3
PUFAs: polyunsaturated fatty acids
DHA: docosahexaenoic acid
EPA: eicosapentaenoic acid
HBsAg: hepatitis B surface antigen
HCV: hepatitis C virus
QC: quality control
ESI: electrospray ionization
VIP: variable importance in the projection.
