# Supplementary material for: Comparison of hepatic and serum lipid signatures in hepatocellular carcinoma patients leads to the discovery of diagnostic and prognostic biomarkers
Source: Oncotarget. 2017 Dec 13;9(4):5032–43. doi: 10.18632/oncotarget.23494 (PMC5797031; doi:10.18632/oncotarget.23494)
Supplement: Supplementary file 1 [file oncotarget-09-5032-s001.pdf]

Comparison of hepatic and serum lipid signatures in hepatocellular carcinoma patients leads to the discovery of diagnostic and prognostic biomarkers

SUPPLEMENTARY MATERIALS

Semi-quantitative analysis of PEp (36:4) and (40:6) in external validation set

Untargeted analysis is the most commonly used method for biomarker discovery by profiling the whole set of biological entities. Targeted analysis is a gold standard for absolutely quantitative analysis of target biomarkers using standards. In other worlds, the absolutely quantitative analysis of target biomarkers is restricted by the coverage of standards. Generally, it is not possible to obtain a standard for every single biomarker. When commercial standard is not available for target biomarker, semi-quantitative analysis is an alternative approach to be carried out, which assigns approximate measurements to data, rather than an exact measurement. In the validation study, we performed a semi-quantitative analysis of PEp (36:4) and (40:6) in 18 hepatocellular carcinoma patients, 20 liver cirrhosis patients and 20 healthy individuals because of no available standards, in which the precursor and product ion pairs (i.e., mass transitions) of the two lipids were defined from the MS/MS study in profiling analysis, the calibration curves were generated by the two lipids from real samples (1, 2).

Chemicals and reagents

Acetonitrile, methanol, and chloroform were purchased from Merck. Ammonium formate and isopropyl alcohol were purchased from Fluka. Deionised water was purified "in-house" using a Milli-Q system Millipore. Lipid standard of PC (17:0/17:0) was purchased from Avanti Polar Lipids.

Sample preparation

Serum (100 µL) was diluted with 900 µL of chloroform/methanol/water (2:1:1, v/v/v) containing 5 µg/mL PC (17:0/17:0) as internal standard. The mixture was shaken vigorously for 5 min, and then centrifuged for 20 minutes at 20,187 g. The organic phase was collected and dried under nitrogen gas. The dry residues were reconstituted in 200 µL chloroform/methanol (2:1, v/v) for LC-MS/MS analysis. The quality control (QC) sample was prepared by mixing an equal aliquot of each serum sample for linearity and repeatability testing, and also for the QC of real sample analysis. For the linearity test, the QC sample was aliquoted into 10, 20, 50, 100, 200, and 400 µL, respectively, and triplicate samples for each volume of serum were prepared. These aliquots were prepared by the same method for real samples. For the repeatability test, 100 µL QC sample were prepared using the same method for real samples, and 10 replicates were prepared.

LC-MS/MS analysis

LC-MS/MS analysis was performed on an Agilent 1290 ultrahigh pressure liquid chromatography system (Waldbronn, Germany) coupled to a 6490 Triple Quadrupole mass spectrometer with iFunnel Technology. Chromatographic separation was achieved using a *phenomenex Kinetex C18* column (50 × 2.1 mm, 2.6 µm) with a flow rate of 0.40 mL/min at 40°C. Gradient conditions were as follows: 0–5 min, 60-85% A; 5–10 min, 85–100% A; 10–25 min, 100% A; 25–26 min, 100–60% A. The mobile phase A was methanol/acetonitrile/isopropyl alcohol (9:4:2, v/v/v) with 20mM $HCOONH_4$, and B was water/isopropyl alcohol (13:2, v/v) with 20 mM $HCOONH_4$. The injected sample volume was 10 µL for each run. PEp (36:4) and (40:6) were ionized in negative mode with following source parameters: drying gas (N2) temperature 200°C with a flow of 14 L/min, nebulizer gas pressure 20 psi, sheath gas temperature 250°C with a flow of 11 L/min, capillary voltage 3,000 V and nozzle voltage 1500 V. Data were acquired in multiple reaction monitoring (MRM) mode (Supplementary Table 7).

Method validation

The proposed method was validated for linearity and repeatability, but not for accuracy, precision and recovery because of no available standards (1). Briefly, calibration curve was constructed from three replicate measurements of six concentrations of PEp (36:4) and (40:6) in QCs. The method showed a good linearity, with $r^2 > 0.99$. The repeatability of their peak areas was calculated as relative standard deviations (RSD). We found that they had an RDS of < 10% among the 10 replicates, indicating the high repeatability of our method.

REFERENCES

1. Chen S, Kong H, Lu X, Li Y, Yin P, Zeng Z, et al. Pseudotargeted metabolomics method and its application in serum biomarker discovery for hepatocellular carcinoma based on ultra high-performance liquid chromatography/triple quadrupole mass spectrometry. Anal Chem. 2013; 85:8326–33.
2. Shao Y, Zhu B, Zheng R, Zhao X, Yin P, Lu X, et al. Development of urinary pseudotargeted LC-MS-based metabolomics method and its application in hepatocellular carcinoma biomarker discovery. J Proteome Res. 2015; 14:906–16.

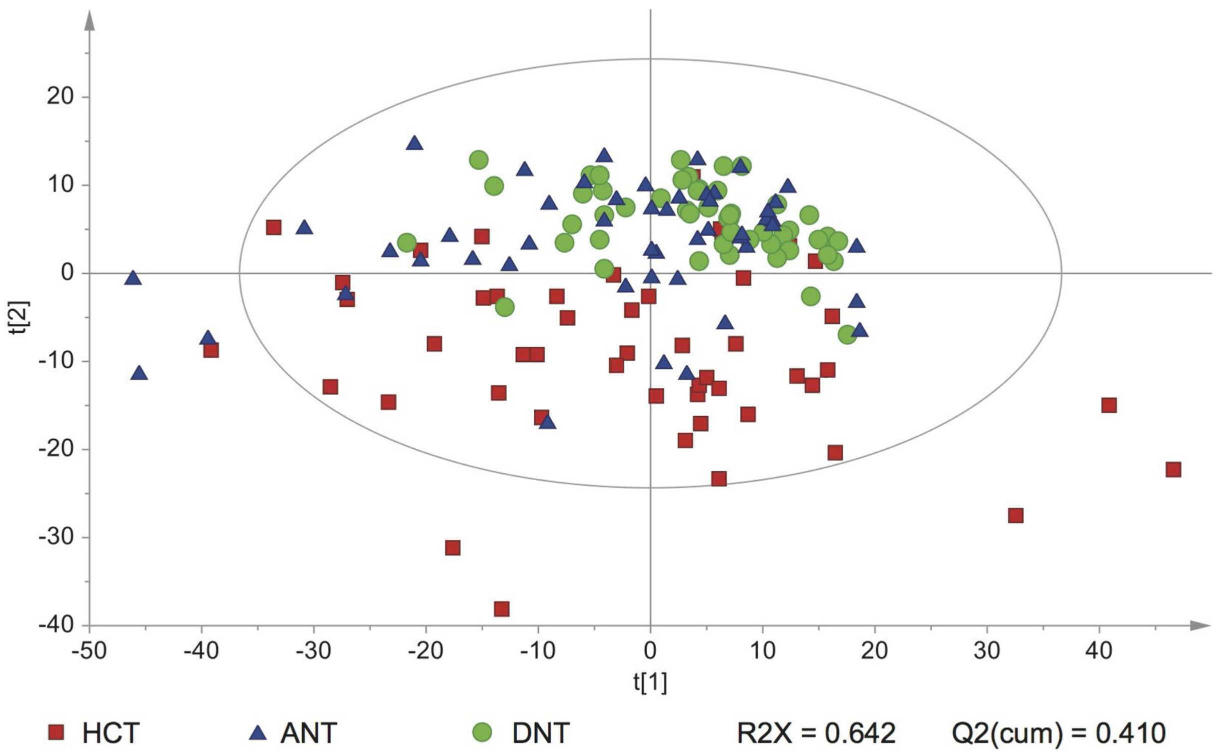

Supplementary Figure 1: PCA score scatter plot shows lipid classification of three types of liver tissues. HCT (n = 50), ANT (n = 50), DNT (n = 50).

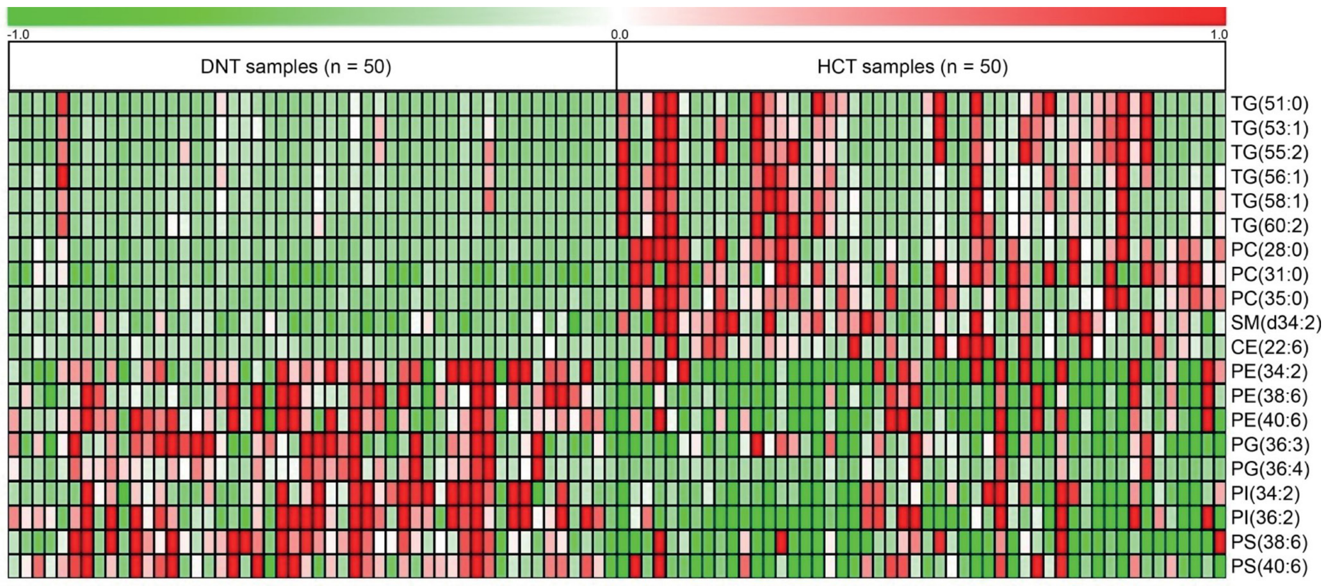

Supplementary Figure 2: Heat map of 20 lipid signatures in DNT and HCT samples. Each row shows peak area for a specific lipid after mean centering and unit variance scaling of the data. Each column shows the hepatic lipid profile of each subject.

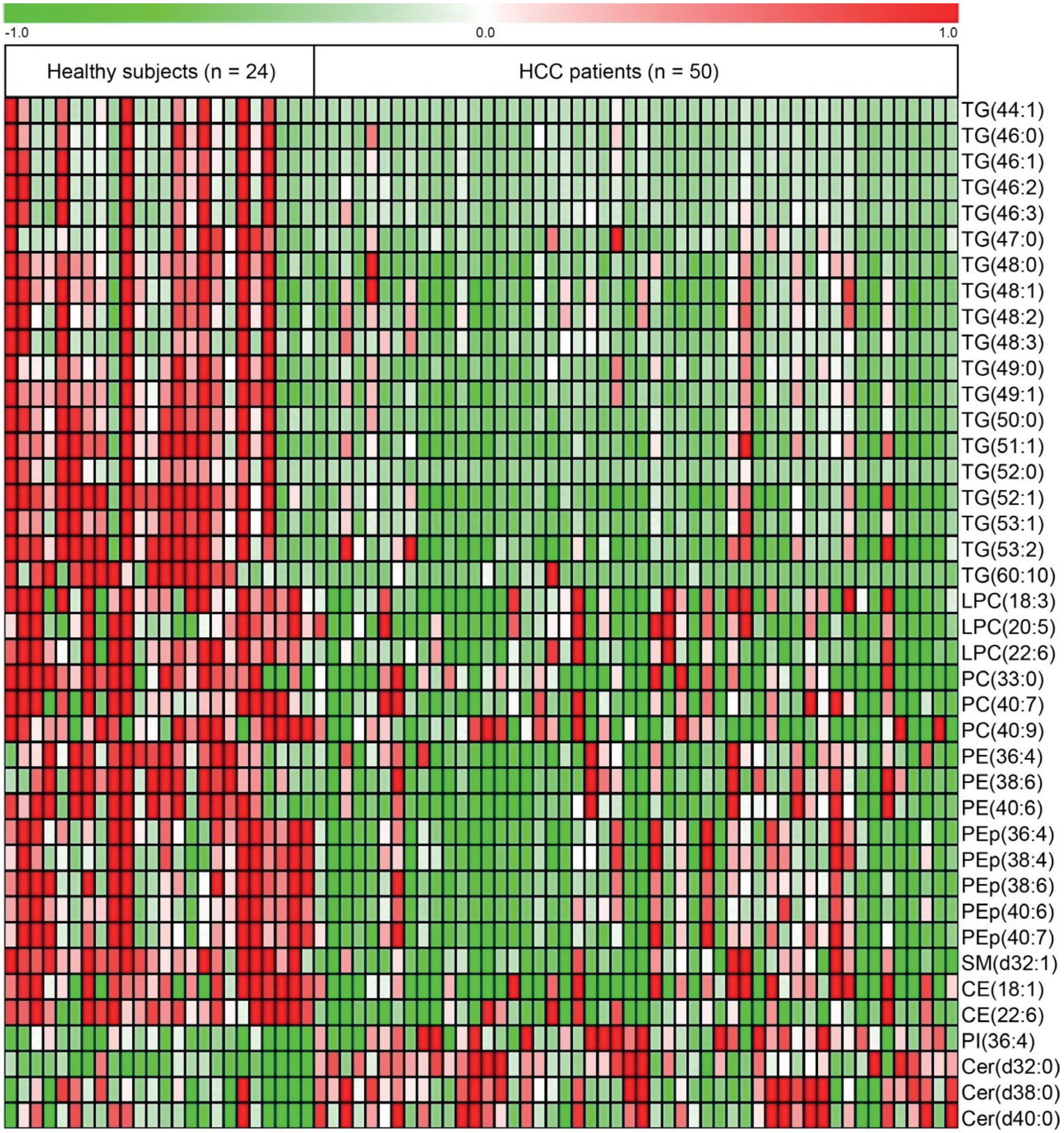

Supplementary Figure 3: Heat map of 40 lipid signatures in serum of healthy subjects and HCC patients. Each row shows peak area for a specific lipid after mean centering and unit variance scaling of the data. Each column shows the serum lipid profile of each subject.

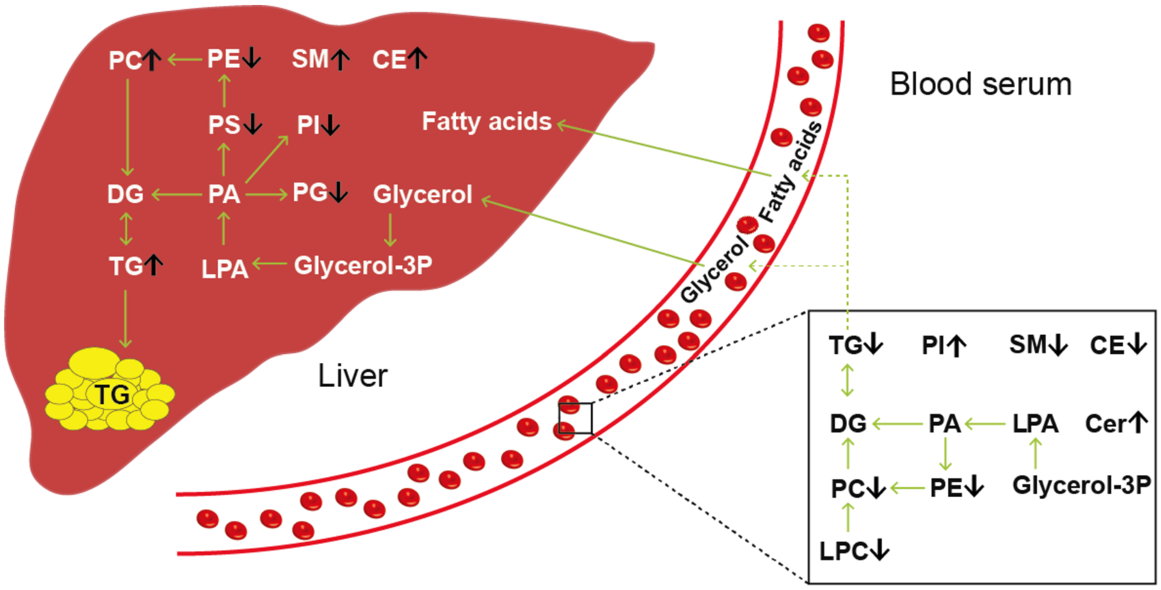

↑ Upregulated in HCC tumors compared to matched normal tissues, or upregulated in HCC sera compared to healthy controls.

↓ Downregulated in HCC tumors compared to matched normal tissues, or downregulated in HCC sera compared to healthy controls.

Supplementary Figure 4: Differential lipids and pathways in liver and serum of HCC patients. Glycerol-3P, Glycerol 3-phosphate.

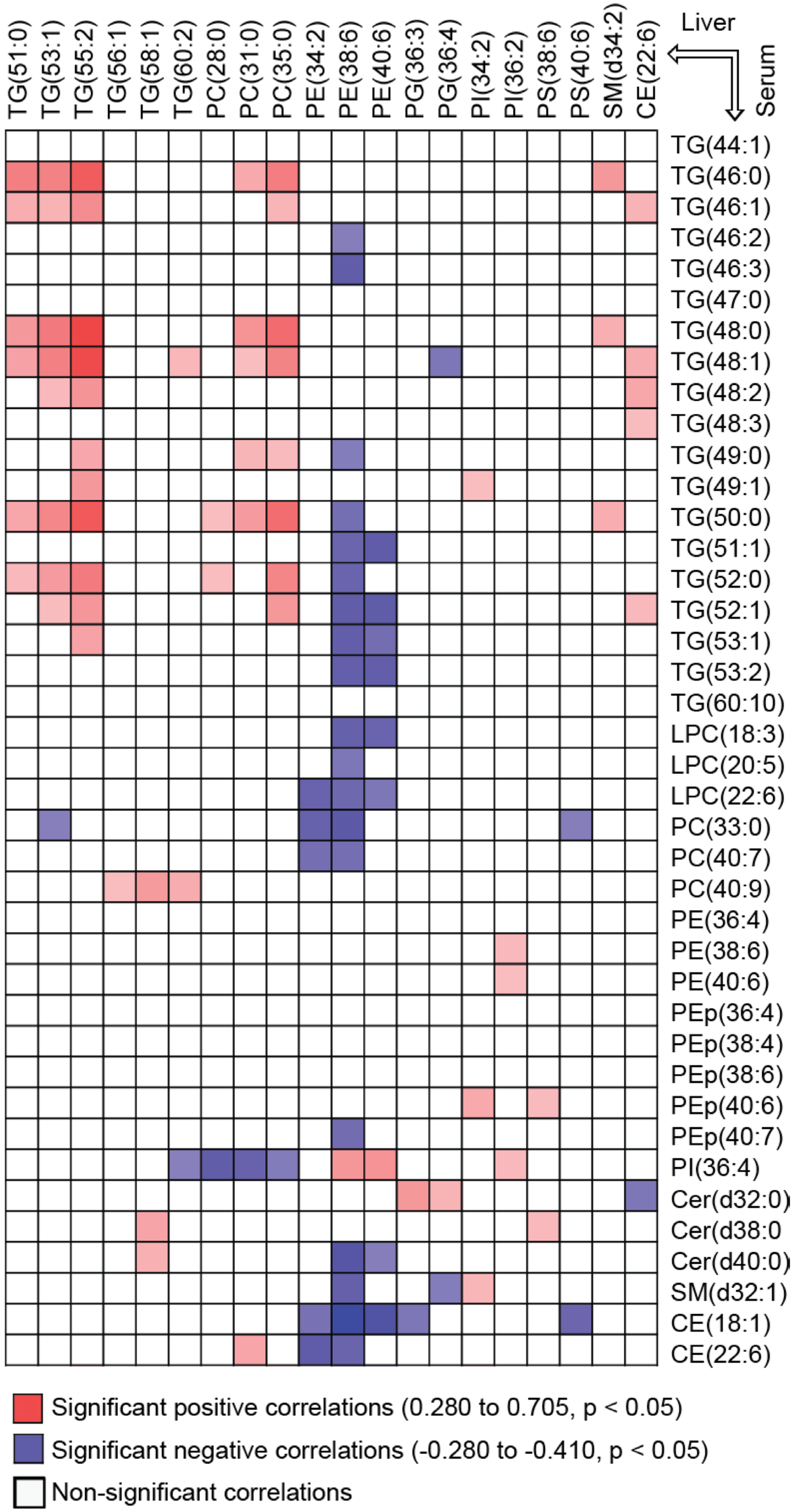

Supplementary Figure 5: Pearson correlation analysis of hepatic and serum lipid changes in HCC patients.

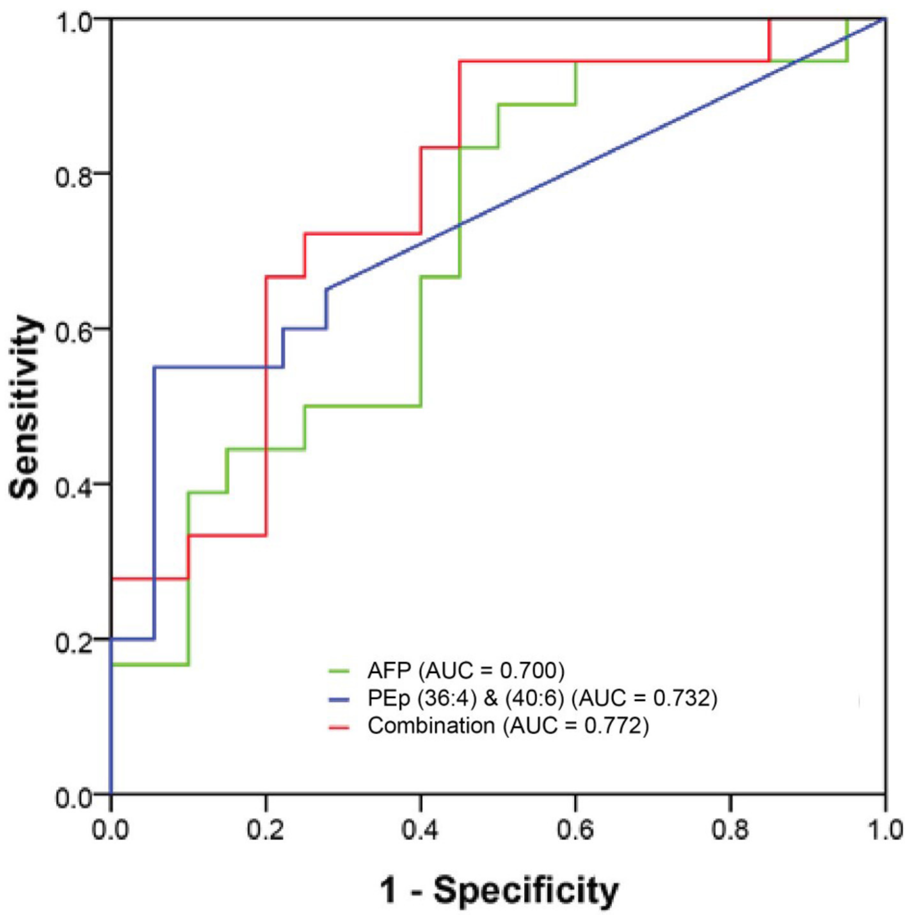

Supplementary Figure 6: ROC curve of the combination of PEp (36:4), (40:6) and AFP to predict HCC from liver cirrhosis patients in the validation set.

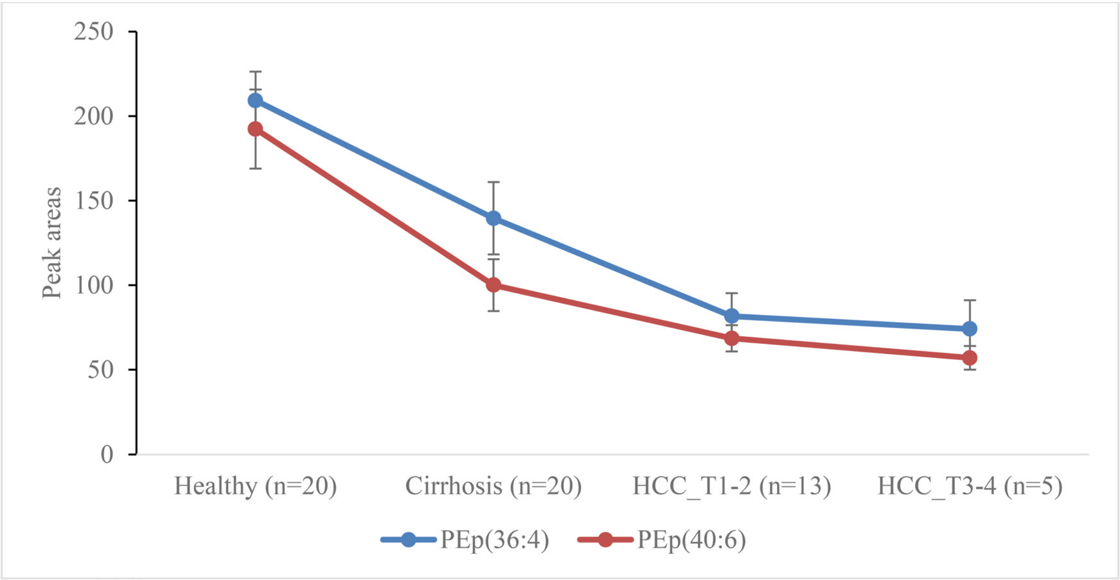

Supplementary Figure 7: Altered serum expressions of PEp (36:4) and (40:6) in the validation set.

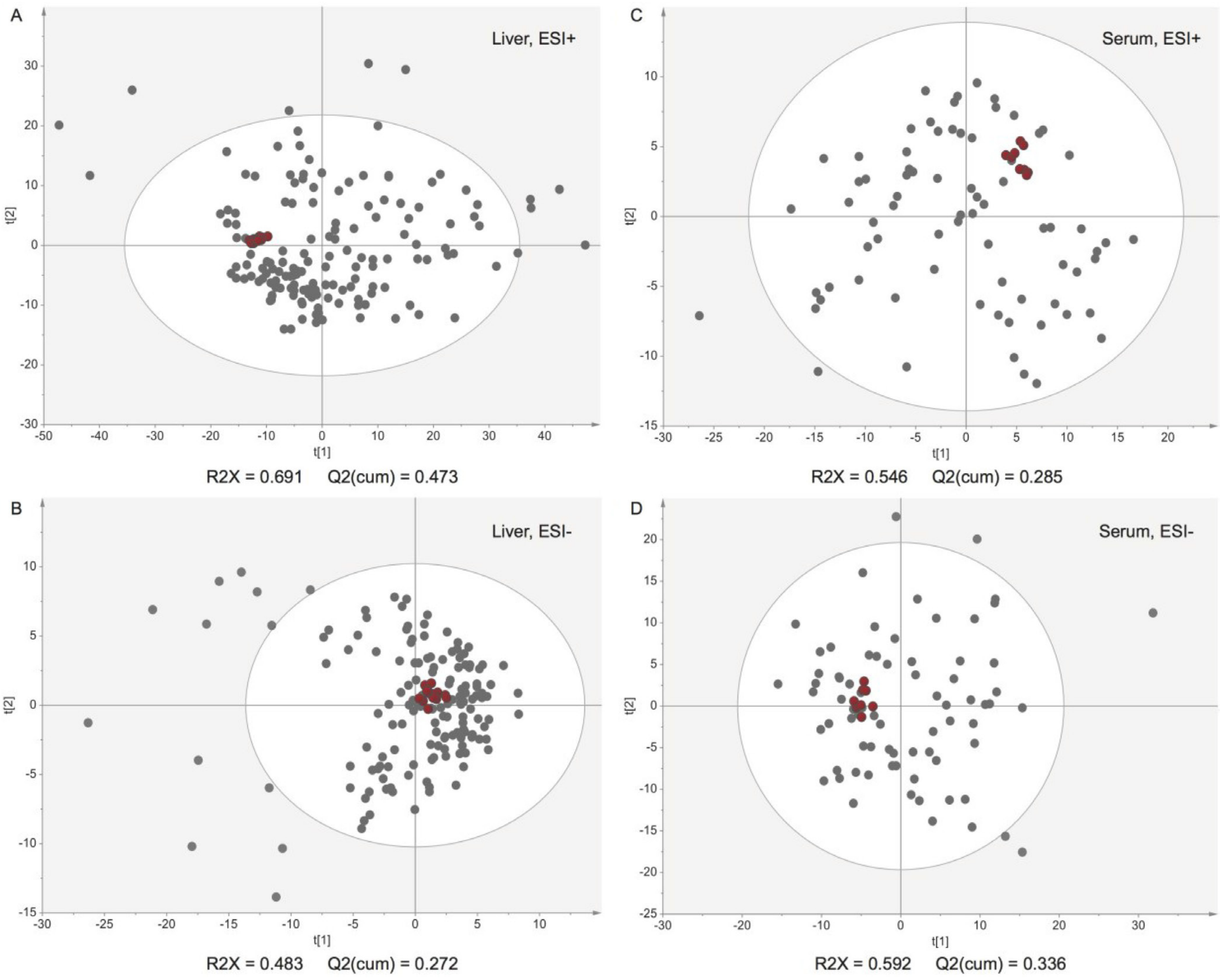

Supplementary Figure 8: PCA scores plots of real (gray dots) and QC (red dots) samples. (**A, B**) tissue (gray dots, *n* = 150) and QC (red dots, *n* = 16) samples: (A) positive ion mode, (B) negative ion mode. (**C, D**) serum (gray dots, *n* = 74) and QC (red dots, *n* = 9) samples: (C) positive ion mode, (D) negative ion mode.

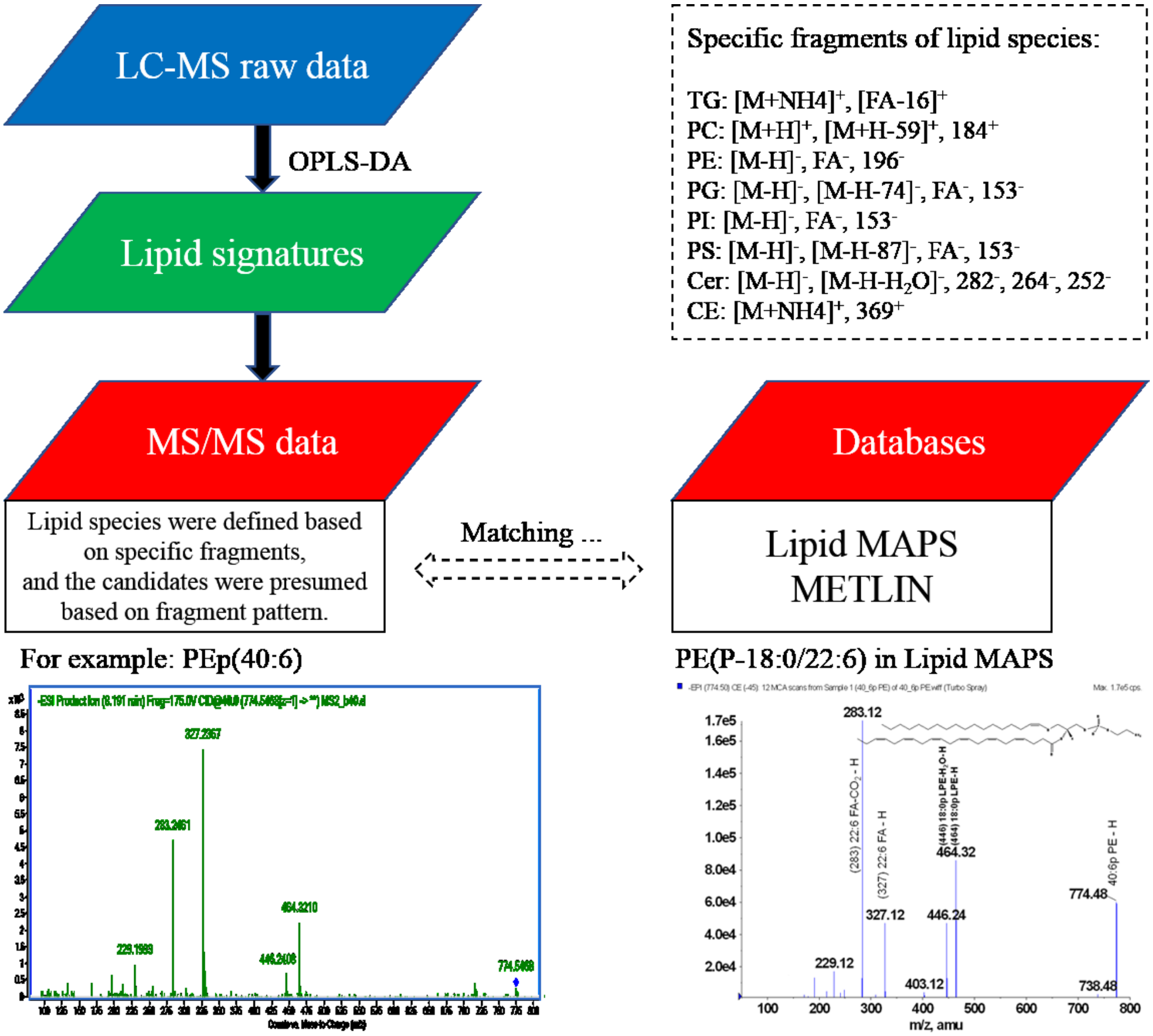

Supplementary Figure 9: A workflow of manual identification of lipids used in this study. TG: triglyceride; PC: phosphatidylcholine; PE: phosphatidylethanolamine; PG: phosphatidylglycerol; PI: phosphatidylinositol; PS: phosphatidylserine; Cer: ceramide; CE: cholesteryl ester; FA: fatty acid.

Supplementary Table 1: Characteristics of 18 HCC patients, 20 liver cirrhosis patients, and 20 healthy individuals in the validation set

Characteristics [a]	HCC patients (n = 18)	Liver cirrhosis patients (n = 20)	Healthy individuals (n = 20)	P value [b]
Gender (M/F)	14/4	13/7	16/4	0.508
Age (year)	60 (43~79)	59 (38~78)	61 (28~69)	0.770
AFP (ng/mL)	7.7 (1.6~1210)	3.0 (1.4~147)	-	0.028
ALT (U/L)	43.5 (29~171)	42.5 (18~495)	-	0.851
AST (U/L)	51.5 (24~181)	57.0 (14~221)	-	0.942
GGT (U/L)	111.9 (24.4~336)	105.9 (15.8~221)	-	0.919
HBsAg (positive/negative)	15/3	14/6	0/24	0.454*
HCV Ab (positive/negative)	3/15	6/14	-	0.454*
Cirrhosis/no cirrhosis	11/7	20/0	0/24	
TNM stages				
T1N0M0 (early-stage)	12			
T2N0M0 (early-stage)	1			
T3N0M0 (late-stage)	4			
T4N0M0 (late-stage)	1			

[a] Age, AFP, ALT, AST, and GGT were expressed as median (range).
[b] Chi-squared test was conducted for gender, HBsAg and HCV Ab differences, one-way ANOVA was for age differences, and Student's t-test was conducted for AFP, ALT, AST, and GGT differences. * The p values are obtained by comparison between HCC and liver cirrhosis patients.

Supplementary Table 2: Characteristics of 50 HCC patients stratified by liver cirrhosis status

Characteristics [a]	HCC patients with liver cirrhosis	HCC patients without liver cirrhosis	P values [b]
No. of subjects	29	21	
Gender (M/F)	23/6	15/6	0.738
Age (year)	53 (34~72)	53 (35~68)	0.317
AFP (mg/L)	49.5 (1.62~24200)	15.5 (3.7~628.6)	0.010
ALT (U/L)	47 (17~444)	94 (22~695)	0.073
AST (U/L)	54 (20~271)	73 (21~903)	0.072
GGT (U/L)	95 (11~305)	61 (17~647)	0.643
HBsAg (positive/negative)	28/1	18/3	
HCVAb (positive/negative)	0/29	1/20	
TNM stages			
T1N0M0 (early-stage)	7	5	
T2N0M0 (early-stage)	11	8	
T3N0M0 (late-stage)	9	1	
T4N0M0 (late-stage)	2	7	

[a] Age, AFP, ALT, AST, and GGT were expressed as median (range). Limitation on the number of subjects, T1 and T2 were set as early-stage of HCC; T3 and T4 were set as late-stage of HCC.
[b] Chi-square test for gender, and Student's t-test for other continuous variables.

Supplementary Table 3: Levels (peak areas) of 20 lipid signatures in HCC tumors stratified by cirrhosis status

Lipids	Candidates	HCC patients without liver cirrhosis (*n* = 21)	HCC patients with liver cirrhosis (*n* = 29)	*P* values [a]
**Glycerolipids**				
TG(51:0)	TG(16:0/17:0/18:0) [iso6]	24.98 ± 6.03	44.67 ± 15.51	0.247
TG(53:1)	TG(16:0/18:1/19:0) [iso6]	65.97 ± 17.75	103.08 ± 40.15	0.405
TG(55:2)	TG(15:0/18:1/22:1) [iso6]	36.39 ± 10.58	53.54 ± 18.00	0.417
TG(56:1)	TG(16:0/18:1/22:0) [iso6]	53.15 ± 12.48	163.03 ± 57.59	0.076
TG(58:1)	TG(16:0/18:1/24:0) [iso6]	30.89 ± 9.92	114.74 ± 39.64	0.052
TG(60:2)	TG(16:0/18:1/26:1) [iso6]	32.33 ± 14.21	75.22 ± 24.13	0.135
**Glycerophospholipids**				
PC(28:0)	PC(14:0/14:0)	10.30 ± 1.38	21.18 ± 7.09	0.147
PC(31:0)	PC(15:0/16:0) [iso2]	8.68 ± 1.52	9.63 ± 2.37	0.738
PC(35:0)	PC(17:0/18:0) [iso2]	6.26 ± 1.24	12.70 ± 4.29	0.163
PE(34:2)	PE(16:0/18:2) [iso2]	856.56 ± 172.28	536.28 ± 161.61	0.182
PE(38:6)	PE(16:0/22:6) [iso2]	1527.88 ± 318.02	763.10 ± 158.95	0.038
PE(40:6)	PE(18:0/22:6) [iso2]	809.98 ± 128.02	544.56 ± 102.39	0.112
PG(36:3)	PG(18:1/18:2) [iso2]	873.24 ± 127.51	811.63 ± 140.54	0.747
PG(36:4)	PG(18:2/18:2)	118.12 ± 29.14	49.46 ± 12.13	0.036
PI(34:2)	PI(16:0/18:2) [iso2]	511.13 ± 81.34	316.20 ± 44.44	0.042
PI(36:2)	PI(18:0/18:2) [iso2]	317.18 ± 50.24	172.01 ± 31.93	0.019
PS(38:6)	PS(16:0/22:6) [iso2]	63.84 ± 16.78	44.33 ± 15.23	0.394
PS(40:6)	PS(18:0/22:6) [iso2]	206.21 ± 31.82	175.13 ± 46.10	0.582
**Sphingolipids**				
SM(d34:2)	SM(d18:1/16:1)	64.54 ± 5.76	107.60 ± 18.44	0.036
**Sterol lipids**				
CE(22:6)		3.50 ± 0.88	5.30 ± 2.47	0.500

[a] Student's *t*-test

Supplementary Table 4: Levels (peak areas) of 40 lipid signatures in HCC serum stratified by cirrhosis status

Lipids	Candidates	HCC patients without liver cirrhosis (n = 21)	HCC patientswith liver cirrhosis (n = 29)	P values [a]
**Glycerolipids**				
TG(44:1)	TG(12:0/14:0/18:1) [iso6]	0.78 ± 0.63	0.63 ± 0.44	0.311
TG(46:0)	TG(12:0/16:0/18:0) [iso6]	1.97 ± 1.02	2.00 ± 1.67	0.945
TG(46:1)	TG(12:0/16:0/18:1) [iso6]	3.45 ± 1.94	3.44 ± 2.16	0.988
TG(46:2)	TG(12:0/16:0/18:2) [iso6]	2.50 ± 1.22	2.64 ± 1.59	0.738
TG(46:3)	TG(12:0/16:0/18:3) [iso6]	1.32 ± 0.76	1.22 ± 0.92	0.689
TG(47:0)	TG(15:0/16:0/16:0) [iso3]	0.44 ± 0.61	0.31 ± 0.35	0.355
TG(48:0)	TG(16:0/16:0/16:0)	9.13 ± 4.06	7.25 ± 4.75	0.150
TG(48:1)	TG(14:0/16:0/18:1) [iso6]	21.32 ± 9.11	19.16 ± 10.65	0.457
TG(48:2)	TG(14:0/16:0/18:2) [iso6]	18.87 ± 8.56	17.3 ± 8.72	0.529
TG(48:3)	TG(14:0/16:1/18:2) [iso6]	5.15 ± 2.82	5.80 ± 4.11	0.538
TG(49:0)	TG(16:0/16:0/17:0) [iso3]	0.72 ± 0.74	0.42 ± 0.52	0.092
TG(49:1)	TG(16:0/16:1/17:0) [iso6]	4.11 ± 1.79	3.39 ± 1.54	0.134
TG(50:0)	TG(16:0/16:0/18:0) [iso3]	8.52 ± 5.45	7.08 ± 4.57	0.317
TG(51:1)	TG(16:0/17:0/18:1) [iso6]	6.36 ± 3.79	5.36 ± 2.56	0.274
TG(52:0)	TG(16:0/18:0/18:0) [iso3]	1.74 ± 1.13	1.46 ± 0.84	0.330
TG(52:1)	TG(16:0/18:0/18:1) [iso3]	57.16 ± 31.95	57.94 ± 22.75	0.921
TG(53:1)	TG(17:0/18:0/18:1) [iso6]	0.70 ± 0.94	0.49 ± 0.62	0.327
TG(53:2)	TG(17:0/18:1/18:1) [iso3]	11.60 ± 6.66	12.03 ± 6.68	0.822
TG(60:10)	TG(18:0/20:4/22:6) [iso6]	0.08 ± 0.07	0.32 ± 0.75	0.158
**Glycerophospholipids**				
LPC(18:3)		12.05 ± 7.73	10.59 ± 7.94	0.520
LPC(20:5)		1.62 ± 2.77	1.58 ± 2.06	0.949
LPC(22:6)		2.09 ± 3.31	2.28 ± 2.03	0.809
PC(33:0)	PC(15:0/18:0) [iso2]	0.74 ± 1.01	1.07 ± 1.09	0.273
PC(40:7)	PC(18:1/22:6) [iso2]	2.99 ± 1.56	3.20 ± 1.70	0.654
PC(40:9)	PC(18:3/22:6) [iso2]	0.54 ± 0.61	0.85 ± 0.60	0.081
PE(36:4)	PE(16:0/20:4) [iso2]	134.50 ± 81.77	99.95 ± 60.60	0.093
PE(38:6)	PE(16:0/22:6) [iso2]	268.83 ± 154.31	172.13 ± 103.38	0.011
PE(40:6)	PE(18:0/22:6) [iso2]	123.51 ± 73.94	79.10 ± 43.81	0.011
PEp(36:4)	PE(P-16:0/20:4)	329.15 ± 208.77	193.66 ± 129.94	0.007
PEp(38:4)	PE(P-18:0/20:4)	473.41 ± 273.53	287.98 ± 174.79	0.005
PEp(38:6)	PE(P-16:0/22:6)	218.28 ± 117.11	149.71 ± 99.25	0.030
PEp(40:6)	PE(P-18:0/22:6)	177.35 ± 105.90	123.17 ± 80.93	0.046
PEp(40:7)	PE(P-18:1/22:6)	121.55 ± 89.00	83.69 ± 69.81	0.099
PI(36:4)	PI(18:2/18:2)	84.82 ± 42.87	67.16 ± 32.22	0.103
Sphingolipids				
**Cer(d32:0)**	Cer(d18:0/14:0)	18.92 ± 11.65	19.89 ± 5.21	0.693
**Cer(d38:0)**	Cer(d18:0/20:0)	0.94 ± 0.38	0.99 ± 0.24	0.535
**Cer(d40:0)**	Cer(d18:0/22:0)	1.16 ± 0.42	1.16 ± 0.29	0.941
**SM(d42:1)**	SM(d18:1/14:0)	19.63 ± 9.54	16.75 ± 6.06	0.200
**Sterol lipids**				
CE(18:1)		0.39 ± 0.35	0.26 ± 0.28	0.150
CE(22:6)		1.47 ± 0.89	1.45 ± 0.97	0.930

[a] Student's t-test

Supplementary Table 5: Associations between serum levels of PEp (36:4) and (40:6) with clinical characteristics in 50 HCC patients (discovery set)

	PEp (36:4)	PEp (40:6)
Age	0.042	0.001
Gender	0.019	0.037
TNM tumor stage	–0.294*	–0.283*
AFP	0.330*	0.234
ALT	–0.168	–0.159
AST	–0.247	–0.184
GGT	–0.183	–0.180

*Correlation is significant at the 0.05 level.

Supplementary Table 6: Associations between serum levels of PEp (36:4) and (40:6) with clinical characteristics in 18 HCC patients (validation set) [a]

	PEp (36:4)	PEp (40:6)
Age	–0.023	0.051
Gender	0.043	–0.189
TNM tumor stage	–0.083	–0.250
AFP	–0.198	–0.120
ALT	–0.029	–0.103
AST	–0.197	–0.039
GGT	0.013	–0.044

[a] No correlation is significant at the 0.05 level.

Supplementary Table 7: Optimized MRM conditions for PEp (36:4) and (40:6)

Compounds	R. T. (min)	Transition ion (m/z)	Fragmentor voltage (V)	CE (eV)
PEp (36:4)	7.91	722.5 –> 436.3<br>722.5 –> 303.2	90	30
PEp (40:6)	8.38	774.5 –> 464.3<br>774.5 –> 327.2	90	30
